# Investigation of the association of weight loss with radiographic hip osteoarthritis in older community-dwelling female adults

**DOI:** 10.1111/jgs.18371

**Published:** 2023-04-19

**Authors:** Zubeyir Salis BEng, Li-Yung Lui, Nancy E. Lane, Kristine Ensrud, Amanda Sainsbury

**Affiliations:** 1 Centre for Big Data Research in Health, The University of New South Wales, Kensington, New South Wales, Australia; 2 San Francisco Coordinating Center, California Pacific Medical Center Research Institute, San Francisco, California, USA; 3 Department of Medicine, School of Medicine, University of California at Davis, Sacramento, California, USA; 4 Department of Medicine and Division of Epidemiology and Community Health, University of Minnesota, Minneapolis, Minnesota, USA; 5 Center for Care Delivery and Outcomes Research, Minneapolis Veterans Affairs Health Care System, Minneapolis, Minnesota, USA; 6 School of Human Sciences, The University of Western Australia, Perth, Western Australia, Australia

**Keywords:** aging, female, hip, osteoarthritis, weight loss

## Abstract

**Objective:**

Most guidelines recommending weight loss for hip osteoarthritis are based on research on knee osteoarthritis. Prior studies found no association between weight loss and hip osteoarthritis, but no previous studies have targeted older adults. Therefore, we aimed to determine whether there is any clear benefit of weight loss for radiographic hip osteoarthritis in older adults because weight loss is associated with health risks in older adults.

**Methods:**

We used data from white female participants aged ≥65 years from the Study of Osteoporotic Fractures. Our exposure of interest was weight change from baseline to follow-up at 8 years. Our outcomes were the development of radiographic hip osteoarthritis (RHOA) and the progression of RHOA over 8 years. Generalized estimating equations (clustering of 2 hips per participant) were used to investigate the association between exposure and outcomes adjusted for major covariates.

**Results:**

There was a total of 11,018 hips from 5509 participants. There was no associated benefit of weight loss for either of our outcomes. The odds ratios (95% confidence intervals) for the development and progression of RHOA were 0.99 (0.92–1.07) and 0.97 (0.86–1.09) for each 5% weight loss, respectively. The results were consistent in sensitivity analyses where participants were limited to those who reported trying to lose weight and who also had a body mass index in the overweight or obese range.

**Conclusion:**

Our findings suggest no associated benefit of weight loss in older female adults in the structure of the hip joint as assessed by radiography.

## INTRODUCTION

Hip osteoarthritis is a disabling joint disease that is highly prevalent in older adults.^1^ By the time they reach 85 years of age, 1 in 4 adults is estimated to be affected by symptomatic hip osteoarthritis.^2^ Hip osteoarthritis significantly reduces disability-adjusted life years.^3^ There is no cure.

For the management of hip osteoarthritis, most guidelines around the world recommend weight loss for people with the condition and concurrent overweight or obesity.^4–9^ However, this recommendation is based on research on people with knee osteoarthritis and overweight or obesity, for whom weight loss brings clear benefits.^10,11^ There is a lack of randomized controlled trials assessing the effectiveness of weight loss for hip osteoarthritis. As the hip joint is less sensitive to obesity and weight change than the knee joint,^12,13^ perhaps related to its ball-and-socket anatomy where mechanical forces are diffused across the joint in comparison to the hinge anatomy of the knee joint,^13–15^ it may be the case that weight loss has no or little benefit for the hip. Indeed, observational studies^16–22^ have not suggested any apparent benefit of weight loss for hip osteoarthritis.

None of the above-mentioned observational studies^16–22^ specifically investigated the effect of weight loss on hip osteoarthritis in older adults, as they involved adults aged between 18 and 79 years. If weight loss has no clear benefit for hip osteoarthritis in older adults, guidelines recommending weight loss for this condition may introduce unnecessary health risks in older adults, for whom weight loss is associated with problems such as increased hip fracture,^23,24^ mortality,^25^ functional impairment, and incident disability.^26^ Therefore, this current study aimed to determine whether weight loss is associated with benefits for structural defects of hip osteoarthritis in older women, using data from the Study of Osteoporotic Fractures (SOF).

## METHODS

### Study design

The SOF is a prospective cohort study of risk factors for osteoporosis and fractures that initially enrolled 9704 white female participants recruited from a population-based listing in four areas of the United States of America.^27^ To be eligible, participants needed to be female, aged 65 years or older at recruitment, and able to walk without assistance, with the exclusion of non-white participants or those who had undergone a bilateral total hip replacement. Ethical approval was obtained by the institutions undertaking the original SOF study, and all participants provided written informed consent.

**FIGURE 1:**
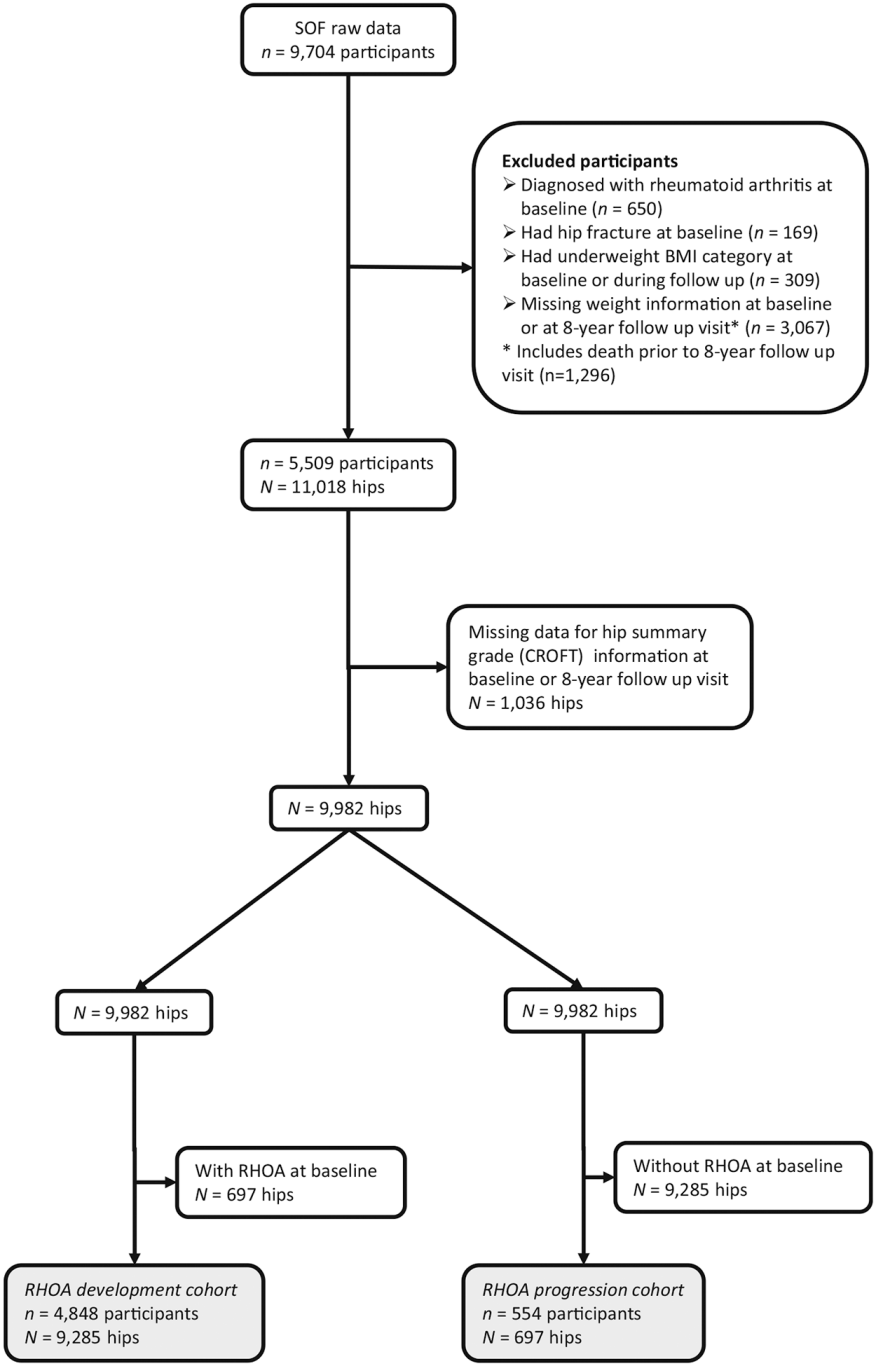
Selection of participants. BMI, body mass index; RHOA, radiographic osteoarthritis; SOF, the study of osteoporotic fractures.

For our sample for the current study, we initially selected those SOF participants who had any data available at either or both baseline (October 1986–October 1988) and the 8-year follow-up (January 1995–June 1996). We then excluded participants with the following characteristics: rheumatoid arthritis at baseline (as assessed by radiography^28^); hip fracture at or before baseline; under-weight body mass index (BMI < 18.5 kg/m^2^) at baseline or at any time point during follow-up (not only at the 8-year follow-up); missing weight data at baseline or at the 8-year follow-up. By excluding participants with missing weight data at the 8-year follow-up visit, we automatically excluded those participants who died before the 8-year follow-up visit (1296 participants). Finally, we excluded hips with missing radiography data at either baseline or the 8-year follow-up visit (Figure 1).

Our exposure of interest was weight change between baseline and the follow-up visit at 8 years (mean [SD] time between baseline and the 8-year follow-up visit was 7.9 [0.4] years), expressed as a percent of baseline weight. Weight change was calculated from the weight of participants which was measured in clinics using balance beam scales.

### Outcomes

We had two primary outcomes related to structural defects of the overall hip as assessed by radiography, as well as nine secondary outcomes related to defects of individual structural features of the hip as assessed by radiography. Our two primary outcomes were the development of radiographic hip osteoarthritis (RHOA) and the progression of RHOA. RHOA was defined as a hip having a modified Croft grade ≥2.^29,30^ The modified Croft grade was rated 0–4 (0: no osteoarthritis; 1: possible osteoarthritis; 2: definite osteoarthritis; 3: moderate osteoarthritis; 4: severe osteoarthritis).^31^ Development of RHOA was defined as a hip having RHOA at the 8-year follow-up visit while not having it at baseline. Progression of RHOA was defined as a hip that had RHOA at baseline having an increase in modified Croft grade ≥1 between baseline and the 8-year follow-up visit.

Our nine secondary outcomes related to defects of individual structural features of the hip were: joint space narrowing (JSN) in the lateral compartment; JSN in the medial compartment; osteophytes on the lateral acetabular surface; osteophytes on the inferior acetabular surface; osteophytes on the lateral femoral surface; osteophytes on the inferior femoral surface; subchondral cysts; subchondral sclerosis; and femoral head deformity. Each defect of an individual structural feature of the hip was rated in one of the following 3 ways: JSN, 0–4; osteophytes, 0–3; and subchondral cysts, subchondral sclerosis, and femoral head deformity, 0 (absent) or 1 (present). Our secondary outcomes were ‘degeneration’ of each of the nine defects of individual structural features of the hip, defined as a change in grade from baseline to the 8-year follow-up visit in one of the following ways: increase in ≥1 grade for JSN in the lateral compartment or osteophytes; increase in ≥2 grades for JSN in the medial compartment (this definition for the degeneration of JSN in the medial compartment is more stringent than that used for the degeneration of JSN in the lateral compartment^32^ due to the high frequency of JSN in the medial compartment in the SOF cohort^30^); or an increase of 1 grade for subchondral cysts, subchondral sclerosis, and femoral head deformity (when it had been grade 0 at baseline).

### Statistical analyses

To investigate our two primary outcomes of the development of RHOA and progression of RHOA, we created 2 cohorts: the ‘RHOA development cohort’ (consisting of hips that did not have RHOA at baseline), and the ‘RHOA progression cohort’ (consisting of hips that had RHOA at baseline) (Figure 1). Our nine secondary outcomes were investigated in both the ‘RHOA development cohort’ and the ‘RHOA progression cohort.’

We used generalized estimating equations with a logistic link function (i.e., logistic regression with clustering of the left and right hip) to estimate the odds ratios (ORs) and 95% confidence intervals (CI) for the association of weight change with our outcomes. Univariate (unadjusted) and multivariable (adjusted) analyses were performed. The multivariable analyses were adjusted for the baseline values of the following 6 variables: age; weight; calcaneal bone mineral density (BMD); use of nonsteroidal anti-inflammatory drugs (NSAIDs); smoking status (never; current; former); and physical activity. In addition, we also adjusted for ‘baseline severity of hip osteoarthritis as assessed by radiography’ (defined as the sum of the modified Croft grade for each of the nine possible defects in individual structural features of the hip, with scores ranging from 0 to 19). These variables were selected because of their association with hip osteoarthritis, as shown in the literature.

In our analyses, weight change was treated as a continuous variable. The assumption of linearity in the association between weight change and our outcomes was tested using the Box-Tidwell method.^33^ There was no violation of this assumption of linearity. We calculated and reported odds ratios for our outcomes based on each 5% weight loss from baseline to the 8-year follow-up visit. We reported the odds ratios based on each 5% weight loss because previous studies suggest that this degree of weight loss is clinically relevant.^18,23,34^ While we report the odds ratios based on each 5% weight loss, we reported the descriptive statistics (i.e., baseline characteristics, and the number of incident cases) stratified by the following three weight change groups of: weight loss (5% or more from baseline to the 8-year follow-up visit); stable weight (less than 5% weight change from baseline to the 8-year follow-up visit); and weight gain (5% or more from baseline to the 8-year follow-up visit).

We did not differentiate between participants who were intentionally trying to lose weight or not, due to the unavailability of this data at baseline. However, data about whether or not participants were intentionally trying to lose weight—regardless of whether or not they did lose weight—was available at the 6-year follow-up visit, and we performed a sensitivity analysis using this data. Here, we explored whether our findings from our main analyses—which included participants with any BMI ≥18.5 kg/m^2^—were consistent with our findings when restricted to only those participants who reported trying to lose weight at the 6-year follow-up visit and who also had a BMI ≥25 kg/m^2^ at baseline (i.e., participants with overweight or obesity). To this end, we created a sub-cohort from the RHOA development cohort and a sub-cohort from the RHOA progression cohort, with each sub-cohort including only participants who answered ‘yes’ to the question of “In the past year, or since you last completed a questionnaire for the study, have you been trying to lose weight?” which was asked at the 6-year follow-up visit, and who also had overweight or obesity at baseline. We then determined the association between weight loss and our outcomes in these 2 sub-cohorts, and compared the results with our main analyses.

We used STATA/BE 17.0 for Windows (64-bit x86–64) for our analyses. We set our threshold for statistical significance as a two-tailed *p* value of less than 0.05.

## RESULTS

### Characteristics of the RHOA development cohort and the RHOA progression cohort

There were 9285 hips from 4848 participants in the RHOA development cohort (Table 1), and 697 hips from 554 participants in the RHOA progression cohort (Table 2). In both cohorts, compared to participants who had a stable weight (less than 5% weight change from baseline) or weight gain (5% or more from baseline), participants who lost weight (5% or more from baseline) tended to be older, heavier, and were more likely to have greater severity of hip osteoarthritis as assessed by radiography at baseline (Tables 1 and 2). In addition, in both cohorts, the majority of participants had a BMI in the overweight or obese category (i.e., BMI ≥25.0 kg/m^2^, 59.5% of participants in the RHOA development cohort, and 55.2% of participants in the RHOA progression cohort) (Tables 1 and 2).

About a third of participants in both cohorts (31.0% in the RHOA development cohort and 32.0% in the RHOA progression cohort) reported that they were trying to lose weight at the 6-year follow-up visit (Supplementary Table S1). About a third of participants in the RHOA development cohort and the RHOA progression cohort (31.1% and 34.5%, respectively) had a weight loss of 5% or more of their baseline weight between baseline and the 8-year follow-up visit. Alternatively, nearly half of the participants (50.4% and 48.4% in the two cohorts, respectively) had maintained their weight within 5% of their baseline weight, and about a fifth of the participants (18.5% and 17.1%, respectively) had a weight gain of 5% or more (Supplementary Table S1). The mean percentage weight change (± SD) in the RHOA development cohort was −1.6 ± 8.6%, and in the RHOA progression cohort it was −2.5 ± 8.9% (Supplementary Table S1). The histograms in Supplementary Figures A and B in the supplementary material show the changes in weight that the hips in both cohorts were exposed to between baseline and the 8-year follow-up.

### Weight loss and the primary outcomes of the development of RHOA and progression of RHOA as assessed by radiography over 8 years

**FIGURE 2:**
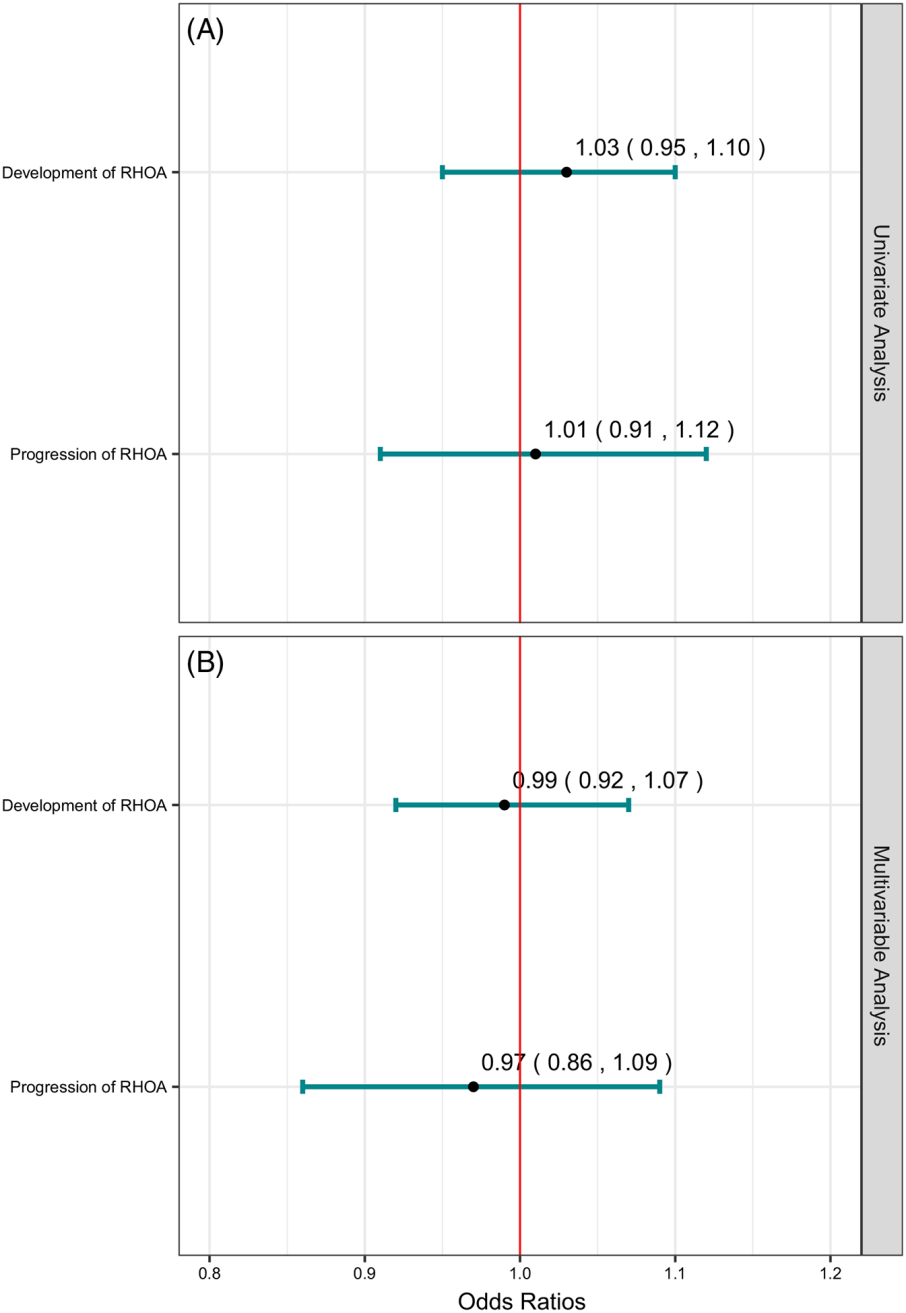
Outcomes of development of RHOA and progression of RHOA. (A). Univariate analysis. (B). Multivariable analysis. The estimates are reported as point estimates of 5% weight loss from baseline to the 8-year follow-up visit. Multivariable analyses were adjusted for the baseline values of age, weight, calcaneus bone mineral density, use of nonsteroidal anti-inflammatory drugs (NSAIDs), smoking status, physical activity status, and the severity of hip osteoarthritis as assessed by radiography (sum of the score from radiography of individual structural features of the hip). RHOA, Radiographic Hip Osteoarthritis.

Of the 9285 hips in the RHOA development cohort (i.e., hips without RHOA at baseline), 292 (3.1%) developed RHOA by the 8-year follow-up visit (Supplementary Table S1). In neither univariate nor multivariable analyses were there any associations of weight loss with the odds of development of RHOA by the 8-year follow-up visit (odds ratio (OR) 1.03 (95% Confidence Interval (CI) 0.95–1.10) and OR 0.99 (95% CI 0.92–1.07 for each 5% weight loss), respectively) (Figure 2).

Of the 697 hips in the RHOA progression cohort (i.e., hips with RHOA at baseline), 146 (21.0%) had progression of RHOA by the 8-year follow-up visit (Supplementary Table S1). In neither univariate nor multivariable analyses were there any associations of weight loss with the odds of progression of RHOA (OR 1.01 [95% CI 0.91–1.12] and OR 0.97 [95% CI 0.86–1.09] for each 5% weight loss, respectively) (Figure 2).

### Weight loss and the secondary outcomes of degeneration of individual structural features of the hip as assessed by radiography over 8 years

The results showed no association of weight loss with the odds of degeneration of any of the nine individual structural features of the hip by the 8-year follow-up visit in the RHOA development cohort or the RHOA progression cohort (Supplementary Figure C).

### Sensitivity analyses

The reader is reminded that our sensitivity analyses were restricted to sub-cohorts of participants who self-reported an intention to lose weight at the 6-year follow-up visit and who had a BMI ≥25 kg/m^2^ at baseline. In the sub-cohort from the RHOA development cohort, 67 (3.1%) of the total of 2176 participants developed RHOA by the 8-year follow-up, while in the sub-cohort from the RHOA progression cohort, 34 (19.3%) of the total of 176 participants had progression of RHOA by the 8-year follow-up. The results of our sensitivity analyses were similar to those from our main analyses, where we included participants regardless of their intention to lose weight and who had a BMI ≥18.5 kg/m^2^ (Supplementary Table S2).

## DISCUSSION

This study found no evidence of association of weight loss with the odds of development or progression of RHOA, or degeneration of any of the nine individual structural features of the hip over 8 years in white female adults aged 65 years or older. Furthermore, findings were consistent when the analyses were restricted to sub-cohorts of participants who self-reported an intention to lose weight and also had a BMI in the overweight or obese range. Thus, weight loss may not be an effective intervention to prevent, slow, or delay radiographic hip osteoarthritis in this population.

These findings add to growing evidence that weight loss has no benefit for hip osteoarthritis. We do not know of any randomized controlled trials that have investigated the effect of weight loss on hip osteoarthritis, but we know of 7 observational studies^16–22^ that investigated the association of weight loss with hip osteoarthritis, in particular for structural defects of the hip joint, hip replacement, and hip pain. Of these 7 studies, 2 studies^18,22^ found no evidence of an association between weight loss and structural defects of the overall hip nor defects in any individual structural features of the hip, and 4 studies^18–21^ found no association with hip replacement (total and/or partial). Of the 4 studies that investigated hip replacement,^18–21^ only one study,^20^ involving participants from the Osteoarthritis Initiative (OAI), followed up over 8 years, showed an association of weight loss with decreased risk of hip replacement, but only in participants who had hip pain at baseline. However, two of the 4 studies that investigated hip replacement^18,21^ included the same OAI cohort in their analyses and did not find any evidence of an association between weight loss and the risk of hip replacement. Of the 7 studies^16–22^ that investigated the association of weight loss with hip osteoarthritis, 3 studies^16–18^ that investigated hip pain showed mixed results, with one^17^ finding a reduction in self-reported hip pain after weight loss, but lacking a control group and a small sample size. None of these seven observational studies^16–22^ specifically investigated the effect of weight loss on hip osteoarthritis in older adults, as they included adults aged between 18 and 79 years. Our current study of older female adults (65 years and older) found no association between weight loss and hip joint structure as assessed by radiography.

While there is no apparent benefit of weight loss for hip osteoarthritis, weight loss is recommended for managing hip osteoarthritis in 6 different health guidelines worldwide.^4–7,9,35^ A notable exception is those from the Osteoarthritis Research Society International guidelines (OARSI),^36^ which cite a lack of clinical trials as a reason for not recommending weight loss for hip osteoarthritis. As mentioned in the introduction section, the recommendation in the guidelines for weight loss in people with hip osteoarthritis is based on research on knee osteoarthritis but not hip osteoarthritis. While weight loss is of benefit for knee osteoarthritis^10,11^ and other aspects of health,^37,38^ it is associated with increased health risks in older adults.^23–26^ Recommending weight loss for people with hip osteoarthritis is sensible if hip osteoarthritis coexists with knee osteoarthritis. However, the guidelines for managing hip osteoarthritis do not specify the requirement of coexistence with knee osteoarthritis. It should be noted that the prevalence of the co-existence of knee and hip osteoarthritis is lower than that of isolated hip osteoarthritis. For example, data from the Canadian Longitudinal Study on Aging (CLSA)^39^ showed that of 1334 people with hip osteoarthritis, 925 (69.3%) had hip osteoarthritis only and 409 (30.7%) had a coexistence of knee and hip osteoarthritis. Given the high prevalence of isolated hip osteoarthritis, combined with the evidence for increased health risks due to weight loss, and no association of weight loss with apparent benefit for hip osteoarthritis, a review of the recommendation of weight loss for the management of hip osteoarthritis in older women who are free of knee osteoarthritis is warranted.

This study has several limitations. Firstly, our findings are associative due to the nature of observational studies. Secondly, there were possibly latent confounders that were not captured in our analyses or the SOF cohort from which we sourced the data for this study. Thirdly, while it is a strength that we studied older adults, our participants were all white, and all female, therefore generalizability of the findings from this study is limited to this population. As a fourth limitation, we measured weight change between baseline and the 8-year follow-up visit, but weight can fluctuate markedly during that time, and we did not capture the possible impact of weight fluctuations on the outcomes in this study. As a fifth limitation, we had no data available to determine whether weight loss was achieved through severe dietary energy restriction or an unbalanced diet that leads to nutritional deficiencies, or whether it was achieved through a balanced diet plus exercise. Given that a balanced diet plus exercise are beneficial for bone health,^40–42^ we cannot rule out the possibility that weight loss achieved through these means would have a positive impact on hip osteoarthritis in older adults. As a sixth limitation, as hip osteoarthritis typically occurs in middle-aged to older adults,^43–45^ our cohort of female adults aged ≥65 years might have been subject to the selection bias of ‘depletion of susceptibles.’^46^ This bias arises when people most susceptible to outcomes of interest (i.e., RHOA) are excluded or unavailable for the study. For example, in the SOF study, people who could not walk without assistance or who had bilateral total hip replacement were excluded. Therefore, our cohort might have consisted of people less susceptible to osteoarthritis, which might have biased our estimates toward the null. As a seventh limitation, this analysis included participants who had to have data on weight and the outcomes of hip osteoarthritis available at the 8-year follow-up and therefore must have survived to the 8-year follow-up. We could not investigate the outcomes for shorter follow-up periods because there was no radiography data at less than an 8-year follow-up. Thus, we can conclude that weight loss is not associated with radiographic hip osteoarthritis among older female adults who survive until an 8-year follow-up. In the general population of older adults, mortality may compete with weight loss with respect to the outcomes of hip osteoarthritis that were investigated in this study. The final limitation of this study is the use of data that was collected over 37 years ago, with baseline data being collected between 1986 and 1988, with an 8-year follow-up. The age of this data may raise concerns about its relevance today due to factors such as changes in medical practices and patient demographics. However, the method used at that time—and in the SOF study—for visualization of hip joint structure (i.e., conventional radiography) is the most commonly-used method today.^47^

In conclusion, there was no associated benefit of weight loss for the structure of the hip as assessed by radiography over 8 years in older adult females. Because previous research has shown potential health risks of weight loss for older adults, our findings call for a review of the recommendation for weight loss for managing hip osteoarthritis for older women who are free of knee osteoarthritis.

## Supplementary Material

Supplementary material **Supplementary Figure A.** Histogram of percent weight change from baseline that hips were exposed to in the RHOA development cohort.
**Supplementary Figure B.** Histogram of percent weight change from baseline that hips were exposed to in the RHOA progression cohort.
**Supplementary Figure C.** Outcomes of degeneration of individual structural features of the hip in univariate and multivariable analyses.
**Supplementary Table S1.** Characteristics of the RHOA development cohort and the RHOA progression cohort during follow-up, stratified by weight loss (5% or more from baseline), stable weight (less than 5% weight change from baseline), and weight gain (5% or more from baseline).
**Supplementary Table S2.** Sensitivity Analyses. Association of weight loss with the odds of development and progression of RHOA by the 8-year follow-up visit in people who had the intention to lose weight and had overweight or obesity, as shown in univariate and multivariable analyses.
**Supplementary STROBE Statement.** Checklist of items that should be included in reports of observational studies.

## Figures and Tables

**TABLE 1:** Baseline characteristics of participants in the RHOA development cohort, stratified by weight loss (5% or more from baseline), stable weight (less than 5% weight change from baseline), and weight gain (5% or more from baseline).

RHOA development cohort					
Characteristics	Weight loss (5% or more from baseline)	Weight stable (less than 5% weight change from baseline)	Weight gain (5% or more from baseline)	Total	*p* value
Participants	*n* = 1510 (31.1)	*n* = 2442 (50.4)	*n* = 896 (18.5)	*n* = 4848 (100.0)	-
Hips	*N* = 2882 (31.0)	*N* = 4682 (50.4)	*N* = 1721 (18.5)	*N* = 9285 (100.0)	
Age, years	71.8 ± 4.9	70.2 ± 4.3	69.2 ± 3.8	70.5 ± 4.5	<0.01
Weight, kg	69.6 ± 12.0	67.1 ± 11.2	66.6 ± 10.3	67.8 ± 11.3	<0.01
Calcaneal BMD, gm/cm^2^	0.41 ± 0.09	0.42 ± 0.09	0.42 ± 0.08	0.41 ± 0.09	< 0.01
NSAID use					0.10
Yes	78 (5.2)	126 (5.2)	31 (3.5)	235 (4.9)	-
No	1425 (94.8)	2303 (94.8)	864 (96.5)	4592 (95.1)	-
Smoking status			-		<0.01
Never smoked	971 (64.5)	1516 (62.2)	531 (59.5)	3018 (62.4)	
Past smoker	421 (28.0)	756 (31.0)	262 (29.3)	1439 (29.8)	-
Current smoker	113 (7.5)	164 (6.7)	100 (11.2)	377 (7.8)	-
Physical activity past week			-		<0.01
Yes	1010 (67.0)	1830 (75.0)	671 (75.1)	3511 (72.5)	
No	498 (33.0)	611 (25.0)	223 (24.9)	1332 (27.5)	-
BMI category					<0.01
Normal (18–<25 kg/m^2^)	500 (33.1)	1077 (44.1)	388 (43.3)	1965 (40.5)	
Overweight (25–<30 kg/m^2^)	624 (41.3)	940 (38.5)	362 (40.4)	1926 (39.7)	
Obese (30 kg/m^2^ or above)	386 (25.6)	425 (17.4)	146 (16.3)	957 (19.8)	
Severity of hip osteoarthritis as assessed by radiography^a^	0.79 ± 1.0	0.73 ± 0.9	0.72 ± 0.9	0.75 ± 0.9	0.06

*Note*: Data are presented as mean ± standard deviation or count (percentage). The percentage calculations are based on complete cases (i.e., excluding missing values). Chi-square and Kruskal-Wallis test analyses were used for comparisons between weight change groups.

Abbreviations: BMD, bone mineral density; BMI, body mass index; NSAID, nonsteroidal anti-inflammatory drug; RHOA, radiographic hip osteoarthritis.

a Sum of the score from radiography of individual structural features of the hip.

**TABLE 2:** Baseline characteristics of participants in the RHOA progression cohort, stratified by weight loss (5% or more from baseline), stable weight (less than 5% weight change from baseline), and weight gain (5% or more from baseline).

RHOA progression cohort					
Characteristics	Weight loss (5% or more from baseline)	Weight stable (less than 5% weight change from baseline)	Weight gain (5% or more from baseline)	Total	*p* value
Participants	*n* = 191 (34.5)	*n* = 268 (48.4)	*n* = 95 (17.1)	*n* = 554 (100.0)	-
Hips	*N* = 244 (35.0)	*N* = 334 (47.9)	*N* = 119 (17.1)	*N* = 697 (100.0)	
Age, years	73.2 ± 5.5	71.5 ± 4.7	69.8 ± 4.2	71.8 ± 5.1	<0.01
Weight, kg	71.7 ± 11.9	67.9 ± 10.7	66.0 ± 9.7	68.9 ± 11.1	< 0.01
Calcaneal BMD, gm/cm^2^	0.42 ± 0.09	0.42 ± 0.09	0.43 ± 0.09	0.42 ± 0.09	0.41
NSAID use					0.67
Yes	13 (6.8)	17 (6.4)	4 (4.2)	34 (6.2)	
No	177 (93.2)	248 (93.6)	91 (95.8)	516 (93.8)	
Smoking status			-		0.09
Never smoked	131 (69.3)	161 (60.1)	58 (61.1)	350 (63.4)	
Past smoker	44 (23.3)	87 (32.5)	26 (27.4)	157 (28.4)	-
Current smoker	14 (7.4)	20 (7.5)	11 (11.6)	45 (8.2)	-
Physical activity past week			-		<0.01
Yes	123 (64.7)	205 (76.5)	81 (85.3)	409 (74.0)	
No	67 (35.3)	63 (23.5)	14 (14.7)	144 (26.0)	-
BMI category					< 0.01
Normal (18–<25 kg/m^2^)	45 (23.6)	101 (37.7)	47 (49.5)	193 (34.8)	
Overweight (25–<30 kg/m^2^)	83 (43.4)	120 (44.8)	35 (36.8)	238 (43.0)	
Obese (30 kg/m^2^ or above)	63 (33.0	47 (17.5)	13 (13.7)	123 (22.2)	
Severity of hip osteoarthritis as assessed by radiography^a^	4.8 ± 2.8	4.4 ± 2.7	3.9 ± 1.7	4.5 ± 2.6	0.03

*Note*: Data are presented as mean ± standard deviation or count (percentage). The percentage calculations are based on complete cases (i.e., excluding missing values). Chi-square and Kruskal-Wallis test analyses were used for comparisons between weight change groups.

Abbreviations: BMD, bone mineral density; BMI, body mass index; NSAID, nonsteroidal anti-inflammatory drug; RHOA, radiographic hip osteoarthritis.

a Sum of the score from radiography of individual structural features of the hip.

## Key points

- As assessed by radiography, there is no associated benefit of weight loss in older female adults over 8 years in improving the structure of the hip joint.

## Why does this paper matter?

Due to the lack of association of weight loss with apparent benefits for hip osteoarthritis, at least for older female adults, this paper calls for a review of the guidelines recommending weight loss for the management of hip osteoarthritis in older adults who are free of knee osteoarthritis.
