# Supplementary material for: Investigation of the association of weight loss with radiographic hip osteoarthritis in older community-dwelling female adults
Source: J Am Geriatr Soc. Author manuscript; Available in PMC 2023 Nov 4. (PMC10624600; doi:10.1111/jgs.18371)
Supplement: Supplementary material — Supplementary Figure A. Histogram of percent weight change from baseline that hips were exposed to in the RHOA development cohort. Supplementary Figure B. Histogram of percent weight change from baseline that hips were exposed to in the RHOA progression cohort. Supplementary Figure C. Outcomes of degeneration of individual structural features of the hip in univariate and multivariable analyses. Supplementary Table S1. Characteristics of the RHOA development cohort and the RHOA progression cohort during follow-up, stratified by weight loss (5% or more from baseline), stable weight (less than 5% weight change from baseline), and weight gain (5% or more from baseline). Supplementary Table S2. Sensitivity Analyses. Association of weight loss with the odds of development and progression of RHOA by the 8-year follow-up visit in people who had the intention to lose weight and had overweight or obesity, as shown in univariate and multivariable analyses. Supplementary STROBE Statement. Checklist of items that should be included in reports of observational studies. [file NIHMS1941004-supplement-Supplementary_material.pdf]

Investigation of Association of Weight Loss with Radiographic Hip Osteoarthritis in Older Community-Dwelling Female Adults

Zubeyir Salis*[1], BEng, Li-Yung Lui[2], MA MS, Nancy E. Lane[3], MD, Kristine Ensrud[4,5], MD MPH, Amanda Sainsbury[6], PhD

1. The University of New South Wales, Centre for Big Data Research in Health, Kensington, NSW, Australia

2 San Francisco Coordinating Center, California Pacific Medical Center Research Institute, San Francisco, California

3. Department of Medicine, University of California at Davis, School of Medicine, Sacramento, California. 95618

4. Department of Medicine and Division of Epidemiology and Community Health, University of Minnesota, Minneapolis MN

5. Center for Care Delivery and Outcomes Research, Minneapolis Veterans Affairs Health Care System, Minneapolis MN

6. The University of Western Australia, School of Human Sciences, Perth, WA, Australia

SUPPLEMENTARY MATERIAL

Contents

Figures ... 3

Figure A. Histogram of percent weight change from baseline that hips were exposed to in the RHOA development cohort ... 3

Figure B. Histogram of percent weight change from baseline that hips were exposed to in the RHOA progression cohort ... 3

Figure C. Outcomes of degeneration of individual structural features of the hip in univariate and multivariable analyses ... 4

Table S1. Characteristics of the RHOA development cohort and the RHOA progression cohort during follow up , stratified by weight loss (5% or more from baseline), stable weight (less than 5% weight change from baseline) and weight gain (5% or more from baseline). ... 5

Table S2. Sensitivity Analyses. Association of weight loss with the odds of development and progression of RHOA by the 8-year follow up visit in people who had the intention to lose weight and overweight or obesity, as shown in univariate and multivariable analyses. ... 7

STROBE Statement. Checklist of items that should be included in reports of observational studies. 8

Figures

Figure A. Histogram of percent weight change from baseline that hips were exposed to in the RHOA development cohort

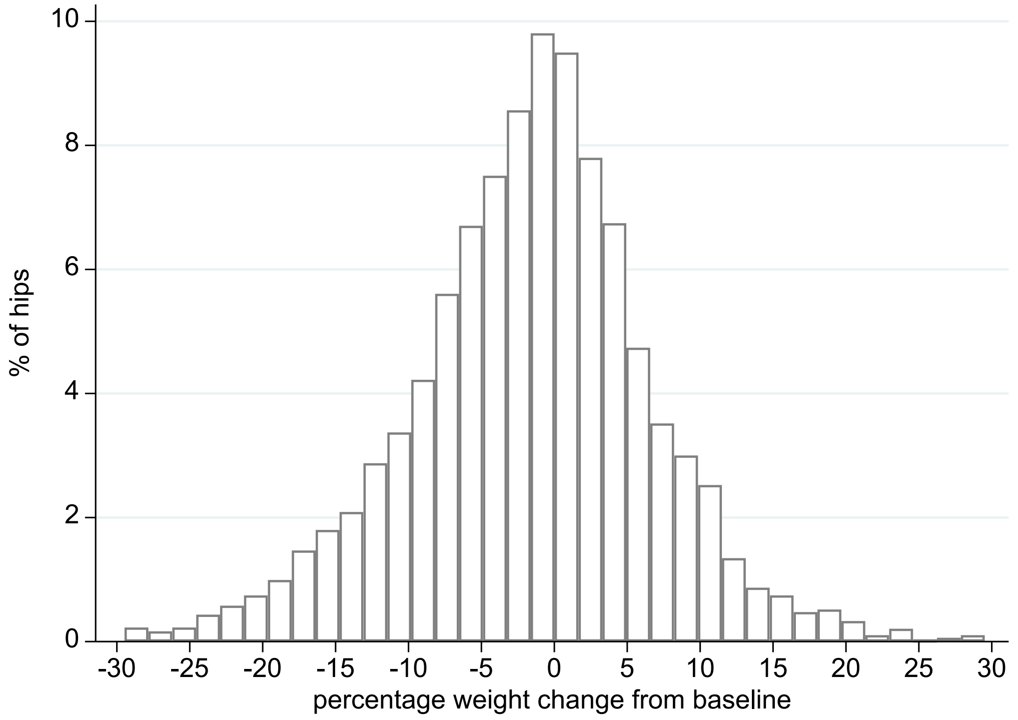

Figure B. Histogram of percent weight change from baseline that hips were exposed to in the RHOA progression cohort

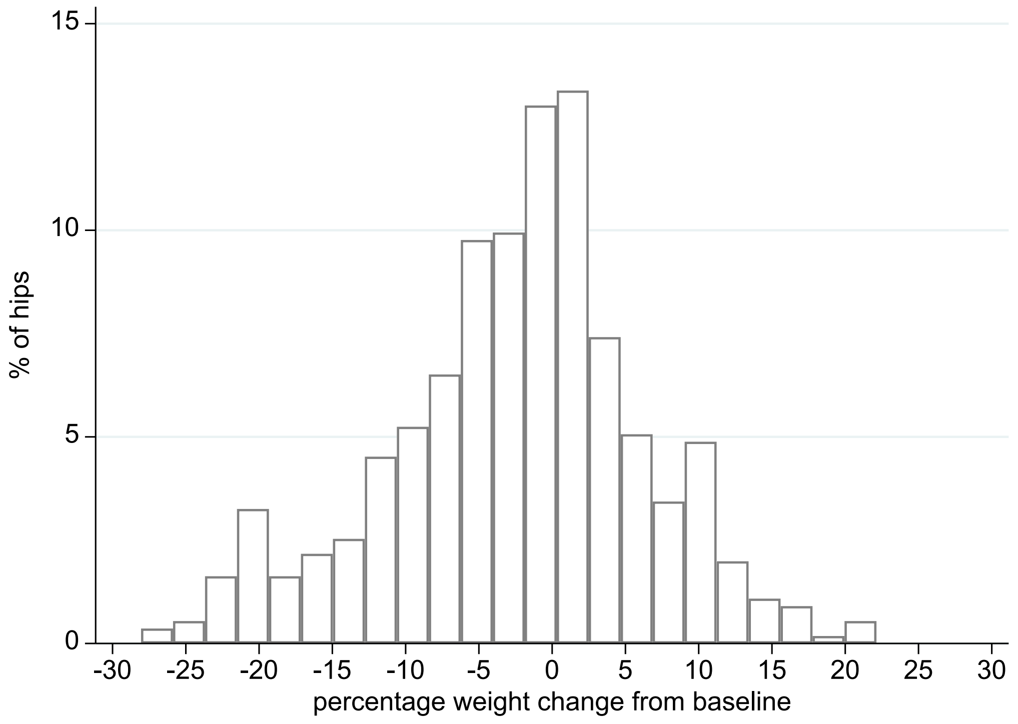

Figure C. Outcomes of degeneration of individual structural features of the hip in univariate and multivariable analyses

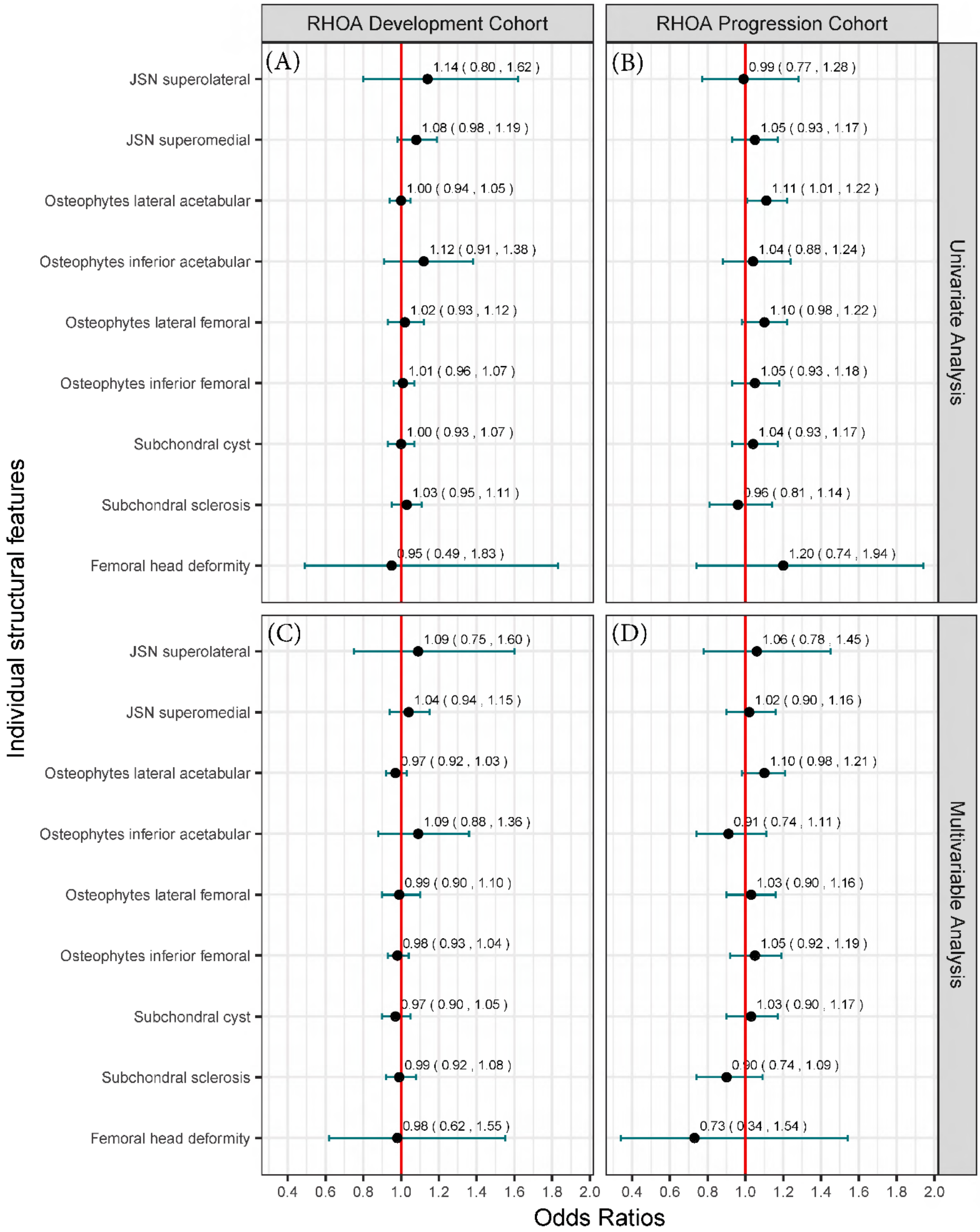

(A). Univariate analysis in RHOA Development Cohort. (B). Univariate analysis in RHOA Progression Cohort. (C). Multivariable analysis in RHOA Development Cohort. (D). Multivariable analysis in RHOA Progression Cohort. The estimates are reported as point estimates of 5% weight loss from baseline to the 8-year follow up visit. Multivariable analyses were adjusted for the baseline values of age, weight, calcaneus bone mineral density, use of nonsteroidal anti-inflammatory drugs (NSAIDs), smoking status, physical activity status, and the severity of hip osteoarthritis as assessed by radiography (sum of the score from radiography of individual structural features of the hip). JSN, Joint Space Narrowing; RHOA, Radiographic Hip Osteoarthritis.

Table S1. Characteristics of the RHOA development cohort and the RHOA progression cohort during follow up , stratified by weight loss (5% or more from baseline), stable weight (less than 5% weight change from baseline) and weight gain (5% or more from baseline).

	**Weight loss (5% or more from baseline)**	**Weight stable (less than 5% weight change from baseline)**	**Weight gain (5% or more from baseline)**	**Total**	**P-value**
RHOA development cohort					
**Participants**	***n* = 1510 (31.1)**	***n* = 2442 (50.4)**	***n* = 896 (18.5)**	***n* = 4848 (100.0)**	**-**
Intention to weight loss^					**< 0.01**
Trying to lose weight	359 (24.2)	744 (30.9)	379 (43.1)	1,482 (31.0)	-
Not trying to lose weight	1,125 (75.8)	1,667 (69.1)	500 (56.9)	3,292 (69.0)	-
Weight change (%)	-11.1 ± 5.4	-0.1 ± 2.7	10.2 ± 5.3	-1.6 ± 8.6	**< 0.01**
**Hips**	***N* = 2882 (31.0)**	***N* = 4682 (50.4)**	***N* = 1721 (18.5)**	***N* = 9285 (100.0)**	**-**
Incidence of development of RHOA					0.95
Yes	91 (3.2)	149 (3.2)	52 (3.0)	292 (3.1)	-
No	2791 (96.8)	4533 (96.8)	1669 (97.0)	8993 (96.9)	-
RHOA progression cohort					
**Participants**	**n = 191 (34.5)**	**n = 268 (48.4)**	**n = 95 (17.1)**	**n = 554 (100.0)**	**-**
Intention to weight loss^					**0.02**
Trying to lose weight	48 (25.5)	88 (33.3)	38 (41.8)	174 (32.0)	-
Not trying to lose weight	140 (74.5)	176 (66.7)	53 (58.2)	369 (68.0)	-
Weight change (%)	-12.0 ± 5.9	-0.2 ± 2.6	10.0 ± 3.9	-2.5 ± 8.9	**< 0.01**
**Hips**	***N* = 244 (35.0)**	***N* = 334 (47.9)**	***N* = 119 (17.1)**	***N* = 697 (100.0)**	**-**
Incidence of progression of RHOA					0.58
Yes	51 (20.9)	74 (22.2)	21 (17.7)	146 (20.9)	-

	**Weight loss (5% or more from baseline)**	**Weight stable (less than 5% weight change from baseline)**	**Weight gain (5% or more from baseline)**	**Total**	**P-value**
No	193 (79.1)	260 (77.8)	98 (82.3)	551 (79.1)	-

Chi-square test analyses were used for comparisons between weight change groups. ^at 6-year follow up.

RHOA: Radiographic Hip Osteoarthritis

Table S2. Sensitivity Analyses. Association of weight loss with the odds of development and progression of RHOA by the 8-year follow up visit in people who had the intention to lose weight and overweight or obesity, as shown in univariate and multivariable analyses.

Outcome	Univariate analyses		Multivariable analyses*	
	Odds ratio (95% CI)	*P* value	Odds ratio (95% CI)	*P* value
Development of RHOA [1]	0.99 (0.85 to 1.15)	0.89	0.93 (0.80 to 1.09)	0.38
Progression of RHOA [2]	0.87 (0.71 to 1.07)	0.20	0.82 (0.64 to 1.06)	0.13

The estimates are reported as point estimates of 5% weight loss from baseline to the 8-year follow up visit, either [1] in the RHOA development cohort, or [2] in the RHOA progression cohort. *Multivariable analyses were adjusted for the baseline values of age, weight, calcaneus bone mineral density, use of nonsteroidal anti-inflammatory drugs (NSAIDs), smoking status, physical activity status, and severity of hip osteoarthritis as assessed by radiography (sum of the score from radiography of individual structural features of the hip). CI: Confidence Interval; RHOA: Radiographic Hip Osteoarthritis.

STROBE Statement. Checklist of items that should be included in reports of observational studies.

	Item No	Recommendation	Location
**Title and abstract**	1	(*a*) Indicate the study's design with a commonly used term in the title or the abstract	Title, Abstract
		(*b*) Provide in the abstract an informative and balanced summary of what was done and what was found	Abstract
**Introduction**			
Background/rationale	2	Explain the scientific background and rationale for the investigation being reported	Introduction (Paragraphs 1-2)
Objectives	3	State specific objectives, including any prespecified hypotheses	Introduction (Paragraph 3)
**Methods**			
Study design	4	Present key elements of study design early in the paper	Methods (Study Design)
Setting	5	Describe the setting, locations, and relevant dates, including periods of recruitment, exposure, follow-up, and data collection	Methods (Study Design)
Participants	6	(*a*) *Cohort study*—Give the eligibility criteria, and the sources and methods of selection of participants. Describe methods of follow-up<br>*Case-control study*—Give the eligibility criteria, and the sources and methods of case ascertainment and control selection. Give the rationale for the choice of cases and controls<br>*Cross-sectional study*—Give the eligibility criteria, and the sources and methods of selection of participants	Methods (Study Design)
		(*b*) *Cohort study*—For matched studies, give matching criteria and number of exposed and unexposed<br>*Case-control study*—For matched studies, give matching criteria and the number of controls per case	NA
Variables	7	Clearly define all outcomes, exposures, predictors, potential confounders, and effect modifiers. Give diagnostic criteria, if applicable	Methods (sections: Study design Paragraph 3, Outcomes, Statistical Analyses paragraph 2)
Data sources/ measurement	8*	For each variable of interest, give sources of data and details of methods of assessment (measurement). Describe comparability of assessment methods if there is more than one group	Methods (sections: Study design Paragraph 3, Outcomes, Statistical Analyses paragraph 2)
Bias	9	Describe any efforts to address potential sources of bias	Methods (section: Statistical analyses)
Study size	10	Explain how the study size was arrived at	Figure 1 and Methods (Study Design Paragraph 2)
Quantitative variables	11	Explain how quantitative variables were handled in the analyses. If applicable, describe which groupings were chosen and why	NA
Statistical methods	12	(*a*) Describe all statistical methods, including those used to control for confounding	Methods (section: Statistical analyses)
		(*b*) Describe any methods used to examine subgroups and interactions	Methods (section: Statistical analyses)
		(*c*) Explain how missing data were addressed	Methods (Study Design Paragraph 2 and Statistical analyses)
		(*d*) *Cohort study*—If applicable, explain how loss to follow-up was addressed<br>*Case-control study*—If applicable, explain how matching of cases and controls was addressed<br>*Cross-sectional study*—If applicable, describe analytical methods taking account of sampling strategy	Methods (section: Statistical analyses)

		(*e*) Describe any sensitivity analyses	Methods (section: Statistical analyses)

Continued on next page

**Results**			**Page No**
Participants	13*	(a) Report numbers of individuals at each stage of study—eg numbers potentially eligible, examined for eligibility, confirmed eligible, included in the study, completing follow-up, and analysed	Figure 1 and Results (Characteristics of the RHOA development cohort and the RHOA progression cohort).
		(b) Give reasons for non-participation at each stage	N/A
		(c) Consider use of a flow diagram	Figure 1
Descriptive data	14*	(a) Give characteristics of study participants (eg demographic, clinical, social) and information on exposures and potential confounders	Tables 1 and 2, Figures A and B. Results (Characteristics of the RHOA development cohort and the RHOA progression cohort)
		(b) Indicate number of participants with missing data for each variable of interest	Methods (Study Design) and Figure 1
		(c) *Cohort study*—Summarise follow-up time (e.g., average and total amount)	Methods (Study Design)
Outcome data	15*	*Cohort study*—Report numbers of outcome events or summary measures over time	Table S1 and Results (Weight loss and the primary outcomes of development of RHOA and progression of RHOA as assessed by radiography over 8 years)
		Case-control study—Report numbers in each exposure category, or summary measures of exposure	N/A
		Cross-sectional study—Report numbers of outcome events or summary measures	N/A
Main results	16	(*a*) Give unadjusted estimates and, if applicable, confounder-adjusted estimates and their precision (eg, 95% confidence interval). Make clear which confounders were adjusted for and why they were included	Table 3 and Figure 2, Results (s Weight loss and the primary outcomes of development of RHOA and progression of RHOA as assessed by radiography over 8 years)
		(*b*) Report category boundaries when continuous variables were categorized	N/A (no categorization of continuous variables)
		(*c*) If relevant, consider translating estimates of relative risk into absolute risk for a meaningful time period	NA

Other analyses	17	Report other analyses done—eg analyses of subgroups and interactions, and sensitivity analyses	Methods (Statistical analyses), Results (Sensitivity analyses), Table S2
**Discussion**			
Key results	18	Summarise key results with reference to study objectives	Discussion (Paragraph 1)
Limitations	19	Discuss limitations of the study, taking into account sources of potential bias or imprecision. Discuss both direction and magnitude of any potential bias	Discussion (Paragraph 4)
Interpretation	20	Give a cautious overall interpretation of results considering objectives, limitations, multiplicity of analyses, results from similar studies, and other relevant evidence	Discussion (Paragraph 1-3, 5)
Generalisability	21	Discuss the generalisability (external validity) of the study results	Discussion (Paragraph 2-4)
**Other information**			
Funding	22	Give the source of funding and the role of the funders for the present study and, if applicable, for the original study on which the present article is based	Funding sources

*Give information separately for cases and controls in case-control studies and, if applicable, for exposed and unexposed groups in cohort and cross-sectional studies.

Note: An Explanation and Elaboration article discusses each checklist item and gives methodological background and published examples of transparent reporting. The STROBE checklist is best used in conjunction with this article (freely available on the Web sites of PLoS Medicine at http://www.plosmedicine.org/, Annals of Internal Medicine at http://www.annals.org/, and Epidemiology at http://www.epidem.com/). Information on the STROBE Initiative is available at www.strobe-statement.org.

END OF DOCUMENT
